# What is the nature of evidence regarding relationships between urban agriculture and gentrification? A systematic map protocol

**DOI:** 10.1186/s13750-025-00375-4

**Published:** 2025-11-13

**Authors:** Anton Parisi, Beatrice Walthall, Paola Clerino, Paula Firmbach, Monika Onyszkiewicz, José Luis Vicente Vicente

**Affiliations:** 1 Leibniz Centre for Agricultural Landscape Research (ZALF), Müncheberg, Germany; 2 Leibniz Centre for Agricultural Landscape Research (ZALF), Thaer-Institute of Agricultural and Horticultural Sciences, Humboldt-University, Berlin, Germany; 3 AgroParisTech, Paris, France; 4 Prinzessinnen Garten, Berlin, Germany; 5 Fundacja EkoRozwoju, Wrocław, Poland; 6 Institute of Economics, Geography and Demography, Spanish National Research Council (CSIC), Madrid, Spain

**Keywords:** Urban geography, Greening, Displacement, Literature review, Academic discourse

## Abstract

**Background:**

As people work towards environmental sustainability for urban environments and everyday lives, tensions have been seen in different efforts on food, housing, environmental management, urban planning, and many cross-cutting issues touching on multiple aspects of social-ecological systems. Urban agriculture (UA) as one multifaceted, cross-cutting arena, has had one particular tension regarding relationships with housing and the built environment: its gentrification potential. However, different accounts have provided evidence and theorization of gentrification as a possible outcome of UA activities, as a risk for UA initiatives, and showing still other relationships between UA and gentrification. These different accounts may be partially explained by different theoretical engagements with gentrification, as well as multiple activities constituting a broad notion of urban agriculture. An overview of the scholarly work regarding these two topics can provide a starting point for understanding how they have been approached and theoretically engaged together, and demonstrate gaps in dominant academic discourses.

**Methods:**

This research for a systematic mapping of literature seeks to assess the academic work around relationships between urban agriculture and gentrification. The protocol outlines a comprehensive and reliable search and review strategy based on the core components of *urban, agriculture,* and *gentrification* in search strings and inclusion criteria. Texts in English, French, and German will be scanned as historically and currently dominant academic languages, while searching nine bibliographic databases or platforms. The protocol details a data coding strategy for metadata, empirical content, and analytic content. The results are expected to uncover sources of evidence for links between urban agriculture and gentrification, producing interoperable datasets of the evidence base, insights of the overall research landscape, and possibilities to find research gaps.

**Supplementary Information:**

The online version contains supplementary material available at 10.1186/s13750-025-00375-4.

## Background

Our increasingly urban world faces challenges new and old regarding how we work towards an equitable and ecologically sustainable future. Urban agriculture (UA), as an umbrella term for multiple approaches to food production and distribution, has been presented as one potential avenue to organize efforts on a number of political agendas and social concerns. Urban agriculture may lead to positive outcomes for food access, economic development, waste management, quality of life, or other effects, but it may also have undesirable consequences or risks. One such social risk is that urban agriculture may lead to gentrification. That is, the increased desirability associated with proximity to farms and gardens might increase the property value to the point of displacing long-time, lower-income residents of the area. Evidence has come out of research in urban geography to support the idea that UA has led to gentrification by boosting neighborhood attractiveness [14, 22] but the relationship between gentrification and UA is not always so straight forward.

In the discipline of geography (as beyond), urban agriculture appears to be a factor in some cases of gentrification, but other accounts show more complexity in the relationship between these two topics. Sites of UA may simultaneously create gentrification pressure and social/political resistance to it [21, 26, 35]. UA initiatives or greening may be a tool intentionally leveraged by local governments [17], or property developers [8, 25], to garner social and political support for gentrification projects. There are also some cases where gentrification is presumed as a risk, but not fully considered (See: [32]) and others calling for inter- and transdisciplinary research in UA to better understand initiatives as social sites of alterity and potential for political engagement [33].

Some research in other schools of thought and in interdisciplinary research contexts have shown that the relationship between urban agriculture and gentrification manifests in still further ways. While some do support the idea that UA leads to gentrification [6], others have worked toward proposing ideas on how to avoid gentrification in UA or greening initiatives (e.g. [24]). While looking at UA as sites for resistance to gentrification, some have supported this idea [7], or pointed to cases where efforts were insufficient [1]. Moreover, others have found that greening may not present the strongest factor in gentrification [27, 29], that UA may not only contribute to displacement pressure, but be the victim of it [28], that displacement pressure may not be merely physical, but include displacing people from a *sense of place* [10], that some forms of UA do not affect real estate prices as keenly as other forms of greening [5], and that greening itself more broadly may not correlate with gentrification [2–4, 13, 34].

**Fig. 1:**
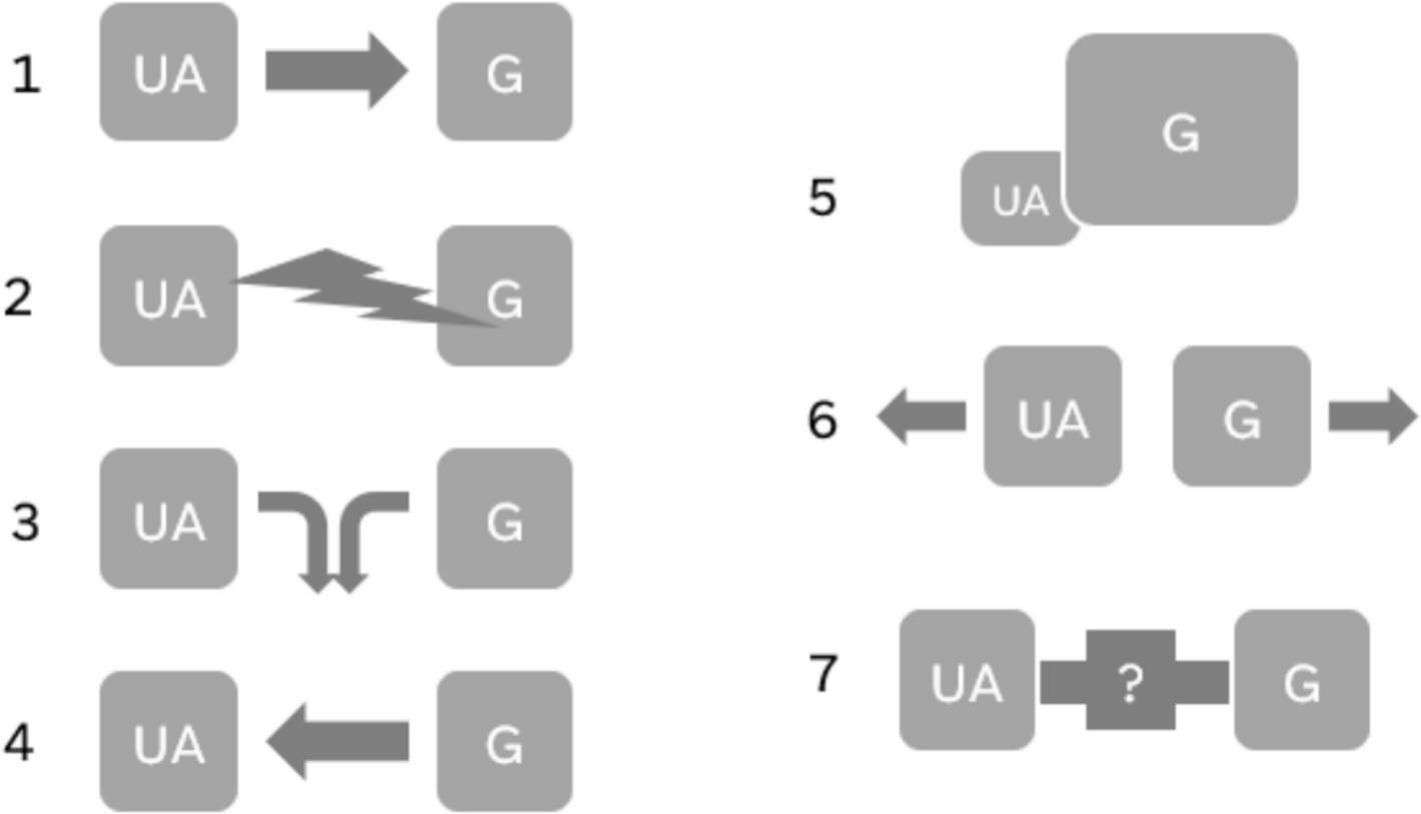
Theoretical model of possible relationships between urban agriculture (UA) and gentrification (G)

Different cases and considerations in literature have been presented regarding the possible relationships between gentrification and urban agriculture. Understanding the relationships between UA and gentrification can help foster social justice, improve informed urban planning, prevent unintended consequences and overall contribute to more nuanced theories and practical frameworks for sustainable urban development. This systematic mapping task seeks to identify the nature of evidence in academic discourse to better understand the sources of evidence and theoretical underpinnings. This method was chosen to give an overview of academic literature in order to identify possible knowledge gaps and collect information about empirical and analytic content while seeking a comprehensive account of the subject topic. Mapping was selected over other evidence synthesis methods (i.e. systematic literature review), as it is understood to be better suited to this aim and broader research questions (here, multiple possible relationships between the principle topics), as opposed to trying to definitively answer a question [15]. The systematic mapping approach is also preferred for resource (time) efficiency, extracting meta-data at the full-text screening stage as opposed to other approaches which may pursue a fuller discourse analysis. This approach will help guide further research, including the possibility of testing academic theory in novel case study areas. The research does not intend to make a generalization about one specific relationship between urban agriculture and gentrification (e.g. urban agriculture leads to gentrification). Some proposed links between gentrification and urban agriculture with different sources of evidence describe different relationships. An initial engagement with the literature has identified some possible patterns across texts for relationships between gentrification and UA which will presumably relate to outputs of this evidence review. This tentative typology is seen in Fig. 1 seven possible relationships as:

1. *UA → Gentrification* (UA leads to gentrification; Aesthetic and economic improvements of a neighborhood increase attractiveness and raises property values, leading to displacement)
2. *UA *Ω *Gentrification* (UA creates sites of social/political resistance to gentrification and displacement)
3. *UA ― Gentrification* (UA and gentrification occur together, but their interaction isn’t known/seen; i.e., correlation without causation)
4. *Gentrification → UA* (Gentrification leads to UA; Neighborhood change leads to greening and food activities)
5. *Gentrification * Δ *UA* (Gentrification displaces UA; farms and gardens face threats to land tenure or access)
6. *UA ≠ Gentrification* (No connection; UA and gentrification share little/no relationship)
7. *UA + Gentrification = ?* (Other connections yet known)

The research topics were explored amongst members of a research project, FOODCITYBOOST, engaging in urban agriculture as well as a small set of actors engaging in the topics of gentrification and/or (peri-) urban agriculture to refine the protocol assumptions and approach. The research questions, sub questions, and search terms were reviewed with actors in gentrification research, agricultural research, a methods specialist, and a judge working in housing law. The protocol authorship is comprised of a team with personal and professional experience in urban agriculture either as a topic of study, an area of employment, or as a non-commercial activity.

### Objective of the review

The systematic map will provide an overview of academic work which shows links between gentrification and urban agriculture. The primary question is: What is the nature of the evidence base for relationships between gentrification and urban agriculture?

The secondary questions are:

1. What is the type of relationship described in the texts?
2. Which factors are believed or evidenced to impact relationship types?
3. What is the type of evidence given?
4. Where are objects of study geographically located?
5. What are the research gaps?

Answering these questions is intended to provide an overview of the different discourses in relation to different evidence bases regarding gentrification. This will help assess what has been suggested to be a key social risk for urban agriculture as it is being pursued to address many ecological and social challenges and goals. It should be noted, however, that the focus of the review yields a sample of texts which consider the topics of gentrification and urban agriculture together, and that cases in which these phenomena are not linked will be necessarily underrepresented in the results.

The research question contains components to consider using the PCC model, namely:

- *Population:* Agricultural initiatives, which are understood as the growing of plants and/or animals for human consumption, livelihood, direct personal use, or collective planting and distribution.
- *Concept:* Relationships with gentrification (e.g. causal, correlational, conditional, coincidental, conflictual, other interactions, none, as outlined in Fig. 1), where gentrification is understood as social and physical changes of an area leading to the displacement of lower-income people and their needs or interests in favor of middle- and upper-class groups. Displacement relating to gentrification is understood in different iterations, as outlined by Marcuse [20]:
  o *Physical displacement* becoming physically unlivable to existing households
  o *Economic displacement* becoming unaffordable to existing households
  o *Exclusionary displacement* housing being unaffordable or unlivable for potential households, not in terms of a whole housing market, but “in a spatially concentrated fashion” (p. 207)
  o *Pressure of displacement* Concern for displacement and physical and economic changes which lead to it, i.e. rise in housing prices and fear of economic displacement
- *Context:* Urban areas, including areas within administrative city limits and metropolitan areas, as well as peri-urban areas.

Definitions for agriculture and urban are based on a UA concept established at the international level beginning around 30 years ago. Supporting current considerations of UA in documents for the European Union and United Nations, these foundational definitions of urban agriculture include the peri-urban, noting physical proximity as well as social proximity, perhaps most distinctly (for agriculture) through supply chains and regional ecology [23, 31]. With an inclusion of growing not only food, but fuels, medicinal plants, and feed for animals, as well as not only growing activities but harvesting and distribution as well (Ibid.), the consideration of agriculture is centered on human management and consumption, separating it from similar activities such as purely aesthetic landscaping. The concept of urban extends past municipal boundaries, with conceptual borders drawn with the city’s “sphere of influence” acting “daily and directly” on the people of the peri-urban area [31], p. 7).

Gentrification is understood as a process of change within a given area with a rich background in academic discourse including its possible causes and iterations in different contexts. This systematic mapping task operationalizes a definition rooted, however, in the empirical. As such, it follows after Marcuse [20] to take gentrification as an observed phenomenon in which established communities "who disproportionately are low-income, working-class and poor, minority and ethnic group members, and elderly" are replaced by newcomers "in a spatially concentrated manner" representing "economic, social, and population changes that cause physical changes to the neighborhoods" (p. 198–99). For this review of literature, the social concern of displacement is the core element of the gentrification concept, while the changes in business profile, demographics, infrastructure, or housing type are possible signals of gentrification in line with Rush Glass’s original description of gentrification [9], as well as current common notions.

## Methods

### Searching for articles

The search terms are grouped into the principal categories: urban, agriculture, and gentrification. Recognizing synonymous terms for each, the lists aim to be extensive without deviating from relevance in the evidence searches. The search terms have been reviewed by actors with experience and expertise in the fields of UA and/or gentrification, including UA practitioners and researchers, and gentrification researchers.

Original search terms for UA and gentrification were created in English (Table 1). Translations in German and French were created and reviewed by fluent speakers with topic expertise. French, German, and English are reviewed as current and historically dominant academic languages. The limitation of not searching in further languages is acknowledged, but the selected languages are considered appropriate to examine the dominant discourses of the Global North/West (with possible texts also from other areas) and should be compared in further study.

**Table 1:** English search terms^1^

Urban	Specifications of farm/garden	Agriculture	Gentrification
Urban* City Cities Metro*	Commun* Allotment* Guerrilla Vertical Indoor Roof* “Zero acreage”	Agricultur* Farm* Garden* “Food forest” Agroforest* Livestock Husbandry Beekeep* Horticultur* Greenhous* Aquaponic* Hydroponic* Aeroponic*	Gentrif* Displac*

The “specifications of farm/garden” group of terms contains modifying terms for those in the Agriculture group (e.g. *community* gardens, *roof-top* greenhouse), and while not synonyms for urban, function as legitimate proxies for urban agriculture in search strings and belong to the same group of terms in the searches.

Any changes to the search strategy will be documented and reported.

^1^ Exact syntax and other search parameters (i.e. date ranges, collections within Web of Science and ProQuest) can be found in the appendix. Search terms in German and French are also included below (Tables 2 and 3).

**Table 2:** French search terms

Urban	Specifications of farm/garden	Agriculture	Gentrification
Urbain* Ville* Métropo*	Communautaire* Collecti* Familia* Insertion* Ouvrier* Partagé* Guérilla Vertical* Indoor Toit* Hors-sol	Agric* Ferme* Jardin* Potager Verger* Agroforest* Élevage Apicult* Apicole* Hortic* Serre Aquaponi* Aquacult* Hydroponi* Aéroponi*	Gentrif* Embourgeoisement Déplac*

**Table 3:** German search terms

Urban	Specifications of farm/garden	Agriculture	Gentrification
Urban* Stadt* Metropo*	Kommun* Kleingarten Kleingärten Schrebergarten Schrebergärten Guerilla* Vertikale* Indoor* Dach*	Landwirt* Anbau* Bauernh* Farm* Garten* Gärten* Lebensmittelwald Agroforst* Vieh* Imker* Biene* Gärtner* Gewächsh* Treibh* Aquaponi* Hydroponi* Hydrokultur Aeroponi*	Gentrif* Verdräng*

Searches will engage nine bibliographic data bases and platforms:

- Scopus
- Lens.org
- AGRIS
- USDA National Agricultural Library (AGRICOLA, PubAg, and NAL Digital Collections)
- AgEcon
- Platforms (databases specified in appendix):
  o Web of Science
  o ProQuest
- Theses/Dissertations:
  o OATD
  o DissOnline (German National LIbrary)

A list of eight texts known to be relevant to the topic through prior reading were assembled as a benchmark standard to assess whether search strings captured the relevant texts [12, 18].

- Fantini, [7]. “Right to the city or environmental gentrification? A discussion about risks and potential of urban agriculture”
- Horst et al., [14]. “The Intersection of Planning, Urban Agriculture, and Food Justice”
- Marche, [19]. “What Can Urban Gardening Really Do About Gentrification”
- McClintock, [22]. “Cultivating (a) Sustainability Capital: Urban Agriculture, Ecogentrification, and the Uneven Valorization of Social Reproduction”
- Sax et al [27, 29] ). “Expelled from the garden? Understanding the dynamics of green gentrification in Vancouver”
- Rosol, [26]. “Politics of urban gardening.” In: *K. Ward, Jonas, A. E. G., Miller, B., Wilson, D. (Eds.): The Routledge Handbook on Spaces of Urban Politics.*
- Sbicca, [30]. “Urban agriculture, revalorization, and green gentrification in Denver, Colorado"
- Wolch et al., [35]. “Urban green space, public health, and environmental justice”

In protocol development, three bibliographic databases/platforms were tested: Scopus, Lens.org, and Web of Science (10 collections of WoS specified within the search string appendix). There were two cases, one in Scopus and one in Lens.org, in which texts are present in the database but did not appear in the search results. The discrepancy was found due to indexing failures, both truncating texts in the abstract field. However, as issues appeared to be only technical and the search strings *did* recover these texts in other databases/platforms, the search strategy was deemed appropriate.

Additionally, citation chasing will be employed in the review of full texts (through Citationchaser, Researchrabbit, or a similar approach) to identify additional texts linking gentrification and UA.

### Article screening strategy and study eligibility criteria

Duplicate texts will be detected first with EndNote21, remaining duplicates with Rayyan and their removal will be documented. Title and abstract screening and proceeding full text screening will be organized on the online platform Rayyan. Four databases offer no or limited ability to export metadata for review: AGRIS, USDA National Agricultural Library, OATD, and DissOnline. For these, screening for duplicate publications and eligibility at the title and abstract stage will be conducted in a web browser.

For all results, a trained review team will scan titles and abstracts for eligibility and document whether texts are irrelevant, documenting the reason for their removal from study (e.g. “displacement” does not refer to people in the text). Consistency checking will be ensured by an initial process of comparing eligibility results by the review team (fuller description below).

In the case that a text lacks an abstract (e.g. book chapters), it will be reviewed in full text screening.

### Eligibility criteria

Eligibility criteria are organized in relation to the PCC model (Population, Concept, and Context) of the research question components (Table 4). Additional criteria related to search parameters are also considered:

- *Language:* Text must be written in English, French, or German as historic and currently dominant academic languages
- *Time frame:* Text written 1964 onward, as the year the term *gentrification* was first published
- *Population:* To verify a text relates to a form or forms of urban agriculture, agriculture must be reasonably determined with people involved in the growing, harvesting, or distribution of plants or animals in food production for human consumption.
- *Concept:* Gentrification potential needs to be determined:
  o If not expressly named, there must be implication of gentrification as: people attracted/migrating to an area, people displaced/moving from an area, land or real estate pricing, change in infrastructure/services, or neighborhood/city character.
- *Context:* Urban areas must be the object of study either by explicit mention of “urban”, “metropolitan”, or “city/cities”, or must relate agriculture with a city’s “sphere of influence” [31] either being within administrative boundaries of a city or metropolitan region, or referring to direct and constant economic influences of the city. While peri-urban also meets inclusion criteria, study of rural areas (with none of the above parameters) would be excluded from study.

**Table 4:** Inclusion and exclusion criteria

Language	Time frame	Urban	Agriculture	Gentrification
Inclusion				
English, French, German	1964 onward	Urban, metropolitan, or city areas written as object of study, including peri-urban, or description of area within administrative boundaries or with direct and constant economic influence of a neighboring city	Agriculture, farming, or gardening written as object of study, or description of plants or animals which are grown for human consumption, livelihood, or harvested and distributed by people	Mention of gentrification, or change of an area in terms of demographics, real estate or rental price, neighborhood or city character, infrastructure, or services which result in displacement or displacement pressure
Exclusion				
Other languages	Before 1964	Study of only rural areas, or no mention of the economic influence of a neighboring city	(Peri-) urban greening only for landscaping purposes, ecological reasons (e.g. reducing heat island effects), or aesthetics (e.g. non-productive green roofs and walls)	No mention of gentrification, AND displacement refers not to people (e.g. water displacement in hydroponic farms) or if so, is not due to changing city or neighborhood character, infrastructure, services, or housing affordability

Following procedural independence, a group of people will be trained for inclusion screening at the title and abstract level to scan 2% of all texts [n = #], alongside the principal reviewer (AP), either in original languages, or from English translations as needed. This process will be repeated as necessary until the level of agreement is determined acceptable, at minimally 95% of articles. Of the articles which remain for full-text screening, 2% of articles [n = #] will be screened by 2 reviewers to check for eligibility criteria. If inclusion agreement is below 95%, differences will be discussed and documented and texts reevaluated before moving to another 2% of texts for review. No reviewers will screen any texts of their own authorship.

### Study validity assessment

A formal validity assessment will not be conducted. Studies which meet the inclusion criteria will not be excluded for low-quality, as the nature of the evidence base is the focus of this systematic mapping exercise. However, information will be extracted which may relate to validity assessment in future reviews:

 :

- Explicit mention of limitations, bias, or alternative interpretations which may relate to validity of the work.
- Citing of an empirical base.
- Evidence type for presence or absence of gentrification (deduction, observation, presumption, none).

### Data coding strategy

The texts from which data will be collected shall be reviewed using a spreadsheet (Excel) to document the meta-data and relevant content. Instructions for each data field (column), as well as dropdown selections for closed-ended fields found in the codebook file, and space for copy-pasted material, included in the appendix. Meta-data to be coded:

### Meta-data

- EPPI ID (via Rayyan)
- Full title
- Author(s)
- Publication year
- Language
- Text type (article, book, chapter, thesis, other)
- Journal (or publisher)

### Empirical content

- Geography of UA/Gentrification
  o Yes (specific location included) / No (theoretical paper: no specific location included)
  o City or cities mentioned (name(s) written)
  o Sub-unit/specific location (name of district, neighborhood, street, or other location such as site of an agricultural initiative)
- Topic-specific content
  o UA type(s) via EFUA typology, [16]: (urban farm, zero acreage farm, social farm, DIY garden/farm, community garden, community park, unclear/unspecified)
  o Gentrification type(s) (Change in the built environment, change in property values, change in housing type, change in commercial/business profile, change in demography, change in location “character”, other changes, no change, physical displacement, economic displacement, exclusionary displacement, displacement pressure)
  o Evidence type for gentrification (deductive: qualitative, quantitative, mixed methods; inductive: observation, presumption/reasoned; none)

### Analytic content

- Relationship type between UA and gentrification (UA leads to G; UA displaced by G; UA resists G; G leads to UA; UA-G concurrent, non-causal; UA-G not connected; Other)
- Factors influencing relationships between UA and gentrification (text passages containing evidence, reason, or suggestions for conditions or phenomena affecting the relationship(s) between UA and G, e.g. type of people involved, policies or regulations, available resources, other influences on property values, acts of resistance, or reasons for the presence, absence, or strength of relationship between UA and gentrification).
- Valuation of gentrification by author or within subjects of research (positive, negative, mixed, unclear)
- Consideration of bias/limitations (text passages explicitly mentioning “limitation”, “bias”, “interpretation” in relation to data collection, analysis, or results)

Consistency for data coding and extraction will be checked for a randomized sample of reviewed texts amounting to 2% [n = x]. Differences will be noted by the principal reviewer and discussed by reviewers.

### Study mapping and presentation

Screening outcomes will be reported according to ROSES standards, using a ROSES flow chart [11] and lists (e.g. spreadsheets) documenting all included texts, as well as those not included, indicating the stage and reason for exclusion.

A geographic projection of the evidence bases will be created using Datawrapper, QGIS, or similar digital program or platform. This will help visualize results pertaining to the fourth research sub-question: “Where is evidence geographically located?” Further maps will use coded data to visualize results pertaining to the first and third sub-questions, “What is the type of relationship described in the texts?” and “What is the type of evidence given?” In this regard, evidence synthesis may also look at relationships of gentrification distinguished by UA type, by valuation of gentrification, and other topic and analytic content if available. Further visualizations will explore number of papers over time (bar graphics), heatmaps, category creation (clustering of the literature), along the lines of the data coded.

To address the fifth and final sub-question, possible knowledge gaps will be identified by first identifying topics or sub-topics in coded data with missing values, and second by noting values which may be over- or under-presented. This will be done by reflecting which (if any) fields (in methodology, geography, topic-specific content, and analytic content) may have few or no results in the reviewed literature, and which (if any) may be the dominating narrative aspects (by comprising, for instance, more than 25% of the texts reviewed).

## Supplementary Information

Supplementary file 1.

Supplementary file 2.

## Declarations

### Data availability

No datasets were generated or analysed during the current study.
