# Supplementary material for: What is the nature of evidence regarding relationships between urban agriculture and gentrification? A systematic map protocol
Source: Environ Evid. 2025 Nov 13;14:22. doi: 10.1186/s13750-025-00375-4 (PMC12616948; doi:10.1186/s13750-025-00375-4)
Supplement: Supplementary file 1 [file 13750_2025_375_MOESM1_ESM.docx]

# Search strings, search parameters, and notes: Systematic map for urban agriculture and gentrification

This document is organized by bibliographic database or platform. Information for each includes exact search strings, search parameters (e.g. search dates, collections, publication date ranges, language, etc.) and notes of information relevant for reproducibility.

This document is to accompany Growing urban change: A systematic map protocol for scanning the relationships between urban agriculture and gentrification, by A. Parisi, B. Walthall, P. Clerino, P. Firmbach, M. Onyszkiewicz, and J. L. Vicente Vicente.

## Scopus

TITLE-ABS-KEY (urban* OR city OR cities OR metro* OR commun* OR allotment* OR guerrilla OR vertical* OR indoor OR roof* OR "zero acreage" OR urbain* OR ville* OR collecti* OR familia* OR insertion* OR ouvrier* OR partage* OR toit* OR hors-sol OR Stadt* OR Kommun* OR Kleingarten OR Schrebergarten OR Guerilla* OR Vertikale* OR Dach*) AND (agric* OR farm* OR garden* OR "food forest" OR agroforest* OR livestock OR husbandry OR beekeep* OR horticultur* OR greenhous* OR aquaponi* OR hydroponi* OR aeroponi* OR ferme* OR jardin* OR potager OR verger* OR elevage OR apicult* OR apicole* OR hortic* OR serre OR Aquacult* OR Landwirt* OR Anbau* OR Bauernh* OR Garten* OR Lebensmittelwald OR Agroforst* OR Vieh* OR Imker* OR Biene* OR Gartner* OR Gewachsh* OR Treibh* OR Hydrokultur) AND (gentrif* OR displac* OR Embourgeoisement OR Deplac* OR Verdrang*)

Parameters: Refine results to date range 1964-2025 and Language limit to English, French, and German.

Search date, number of results: June 11, 2025; **3,180**

## Lens.org

(urban* OR city OR cities OR metro* OR commun* OR allotment* OR guerrilla OR vertical* OR indoor OR roof* OR zero-acreage OR urbain* OR ville* OR collecti* OR familia* OR insertion* OR ouvrier* OR partagé* OR toit* OR hors-sol OR Stadt* OR Kommun* OR Kleingarten OR Schrebergarten OR Guerilla* OR Vertikale* OR Dach*)

AND (agric* OR farm* OR garden* OR food?forest OR agroforest* OR livestock OR husbandry OR beekeep* OR horticultur* OR greenhous* OR aquaponi* OR hydroponi* OR aeroponi* OR ferme* OR jardin* OR potager OR verger* OR Élevage OR apicult* OR apicole* OR hortic* OR serre OR Aquacult* OR Landwirt* OR Anbau* OR Bauernh* OR Garten* OR Lebensmittelwald OR Agroforst* OR Vieh* OR Imker* OR Biene* OR Gärtner* OR Gewächsh* OR Treibh* OR Hydrokultur)

AND (gentrif* OR displac* OR Embourgeoisement OR Déplac* OR Verdräng*)

Parameters: “Scholar Search”; Separate strings in “Title, Abstract, Keyword or Field of Study”

Results refined to date range 1964-2025

Search date, number of results: June 11, 2025; **9,114**

## AGRIS

(urban* *OR* city *OR* cities *OR* metro* *OR* commun* *OR* allotment* *OR* guerrilla *OR* vertical* *OR* indoor *OR* roof* *OR* zero-acreage *OR* urbain* *OR* ville* *OR* collecti* *OR* familia* *OR* insertion* *OR* ouvrier* *OR* partagé* *OR* toit* *OR* hors-sol *OR* Stadt* *OR* Kommun* *OR* Kleingarten *OR* Schrebergarten *OR* Guerilla* *OR* Vertikale* *OR* Dach*) *AND* (agric* *OR* farm* *OR* garden* *OR* food?forest *OR* agroforest* *OR* livestock *OR* husbandry *OR* beekeep* *OR* horticultur* *OR* greenhous* *OR* aquaponi* *OR* hydroponi* *OR* aeroponi* *OR* ferme* *OR* jardin* *OR* potager *OR* verger* *OR* Élevage *OR* apicult* *OR* apicole* *OR* hortic* *OR* serre *OR* Aquacult* *OR* Landwirt* *OR* Anbau* *OR* Bauernh* *OR* Garten* *OR* Lebensmittelwald *OR* Agroforst* *OR* Vieh* *OR* Imker* *OR* Biene* *OR* Gärtner* *OR* Gewächsh* *OR* Treibh* *OR* Hydrokultur) *AND* (gentrif* *OR* displac* *OR* Embourgeoisement *OR* Déplac* *OR* Verdräng*)

Parameters: “Search in [Abstract]”; Language: English, French, or German, Publication date: 1964-2025

Notes: This database to be searched after all others to ease duplication checking, and filtering did not remove all language-irrelevant results

Search date, number of results: June 11, 2025; **1,213**

## USDA NAL

(urban* OR city OR cities OR metro* OR commun* OR allotment* OR guerrilla OR vertical* OR indoor OR roof* OR "zero acreage" OR urbain* OR ville* OR collecti* OR familia* OR insertion* OR ouvrier* OR partagé* OR toit* OR “hors-sol” OR Stadt* OR Kommun* OR Kleingarten OR Schrebergarten OR Guerilla* OR Vertikale* OR Dach*)

AND (agric* OR farm* OR garden* OR "food forest" OR agroforest* OR livestock OR husbandry OR beekeep* OR horticultur* OR greenhous* OR aquaponi* OR hydroponi* OR aeroponi* OR ferme* OR jardin* OR potager OR verger* OR Élevage OR apicult* OR apicole* OR hortic* OR serre OR Aquacult* OR Landwirt* OR Anbau* OR Bauernh* OR Garten* OR Lebensmittelwald OR Agroforst* OR Vieh* OR Imker* OR Biene* OR Gärtner* OR Gewächsh* OR Treibh* OR Hydrokultur)

AND (gentrif* OR displac* OR Embourgeoisement OR Déplac* OR Verdräng*)

Parameters:

- Advanced search; Search fields: “Any field”, “contains”
- Publication date: 1.1.1964 (end date auto-fills 12.31.9999)
- Excluding Subjects: Animals, Geophysics, Temperature
- Language filter of search results: English, French, German

Notes: This database to be searched after all others to ease duplication checking

Search date, number of results: June 11, 2025; **7,601**

## AgEcon

(urban* OR city OR cities OR metro* OR commun* OR allotment* OR guerrilla OR vertical* OR indoor OR roof* OR "zero acreage" OR urbain* OR ville* OR collecti* OR familia* OR insertion* OR ouvrier* OR partagé* OR toit* OR “hors-sol” OR Stadt* OR Kommun* OR Kleingarten OR Schrebergarten OR Guerilla* OR Vertikale* OR Dach*) AND (agric* OR farm* OR garden* OR "food forest" OR agroforest* OR livestock OR husbandry OR beekeep* OR horticultur* OR greenhous* OR aquaponi* OR hydroponi* OR aeroponi* OR ferme* OR jardin* OR potager OR verger* OR Élevage OR apicult* OR apicole* OR hortic* OR serre OR Aquacult* OR Landwirt* OR Anbau* OR Bauernh* OR Garten* OR Lebensmittelwald OR Agroforst* OR Vieh* OR Imker* OR Biene* OR Gärtner* OR Gewächsh* OR Treibh* OR Hydrokultur) AND (gentrif* OR displac* OR Embourgeoisement OR Déplac* OR Verdräng*)

Parameters: Results sorted by date and filtered to exclude publications before 1964 (n=5).

Search date, number of results: June 11, 2025; **98**

## Web of Science

(urban* OR city OR cities OR metro* OR commun* OR allotment* OR guerrilla OR vertical* OR indoor OR roof* OR "zero acreage" OR urbain* OR ville* OR collecti* OR familia* OR insertion* OR ouvrier* OR partagé* OR toit* OR hors-sol OR Stadt* OR Kommun* OR Kleingarten OR Schrebergarten OR Guerilla* OR Vertikale* OR Dach*) AND (agric* OR farm* OR garden* OR "food forest" OR agroforest* OR livestock OR husbandry OR beekeep* OR horticultur* OR greenhous* OR aquaponi* OR hydroponi* OR aeroponi* OR ferme* OR jardin* OR potager OR verger* OR Élevage OR apicult* OR apicole* OR hortic* OR serre OR Aquacult* OR Landwirt* OR Anbau* OR Bauernh* OR Garten* OR Lebensmittelwald OR Agroforst* OR Vieh* OR Imker* OR Biene* OR Gärtner* OR Gewächsh* OR Treibh* OR Hydrokultur) AND (gentrif* OR displac* OR Embourgeoisement OR Déplac* OR Verdräng*)

Parameters:

- Collections (10): Core Collection 1945-present; Biological Abstracts 1926-present; BIOSIS Citation Index 2013-present; BIOSIS Previews 1926-2004; Current Contents 1998-present; CABI: CAB Abstracts 1973-present; Data Citation Index 1994-present; KCI-Korean Journal Database 1980-present; Preprint Citation Index 1991-present; SciELO Citation Index 2002-present;
- Topic searches (Title, abstract and indexing)
- Date range: 1964-01-01 to 2025-06-11
- Preprint Citation Index becomes deselected and must be re-included
- Filter results: English, French, German
- Filter results, exclude Research Areas: Zoology, Veterinary Sciences, Nutrition Dietetics, Meteorology, Physiology, Instruments, Biochemistry, Reproductive Biology, Infectious Diseases, Marine Freshwater Biology, Pathology, Genetics, Energy and Fules, Anatomy, Chemistry, Entomology, Mathematical Computational Biology, Geology, Endocrinology, Gestroenterology, Geochemistry, Evolutionary Biology, Microbiology, Developmental Biology, Surgery, Pharmacology, Oceanography, Parasitology, Physics, Immunology, Cell Biology, Neurosciences, Obstetrics, Orthopedics, Mining, Cardiology, Internal Medicine, Pediatrics, Radiology, Medical Laboratory Technology, Respiratory System, Dermatology, Experimental Medicine, Anesthesiology, Paleontology, Thermodynamics, Virology, Hematology, Mycology, Urology, Mechanics, Archaeology, Microscopy, Optics, Dentistry, Nuclear Science, Tropical Medicine, Spectroscopy, Ophthalmology, Medical Ethics, Oncology, Electrochemistry, Emergency Medicine, Integrative Complementary Medicine, Medical Informatics, Minerology, Metallurgy, Astronomy, Critical Care Medicine, Legal Medicine, Otorhinolaryngology, Crystallography, Polymer Science, Rheumatology, and Transplantation.

Notes: deselect collections: Derwent Innovations index (patents); Grants Index (not publications); FSTA (not relevant topic); ProQuest (separate search) Zoological Record (not relevant topic)

Search date, number of results: June 11, 2025; **1,684**

## ProQuest

Parameters:

- Advanced search, Fields: Anywhere except full text
- Publication date: “After this date…” January 1 1964
- Source types: Books, Conference Papers, Dissertations & Thesis, Scholarly Journals, and Working Papers
- Language: English, French, German
- Databases Searched: African Writers Series, ARTbibliographies Modern for DFG, Coronavirus Research Database, Digital National Security Archive, Early Modern Collection, Early English Books Online, Ebook Central, Gerritsen Women’s History Collection of Aletta H. Jacobs, Literature Online, Periodicals Archive Online, Periodicals Index Online, Publicly Available Content Database)

noft(agric* OR farm* OR garden* OR "food forest" OR agroforest* OR livestock OR husbandry OR beekeep* OR horticultur* OR greenhous* OR aquaponi* OR hydroponi* OR aeroponi* OR ferme* OR jardin* OR potager OR verger* OR Élevage OR apicult* OR apicole* OR hortic* OR serre OR Aquacult* OR Landwirt* OR Anbau* OR Bauernh* OR Garten* OR Lebensmittelwald OR Agroforst* OR Vieh* OR Imker* OR Biene* OR Gärtner* OR Gewächsh* OR Treibh* OR Hydrokultur) AND noft(urban* OR city OR cities OR metro* OR commun* OR allotment* OR guerrilla OR vertical* OR indoor OR roof* OR "zero acreage" OR urbain* OR ville* OR collecti* OR familia* OR insertion* OR ouvrier* OR partagé* OR toit* OR hors-sol OR Stadt* OR Kommun* OR Kleingarten OR Schrebergarten OR Guerilla* OR Vertikale* OR Dach*) AND noft(gentrif* OR displac* OR Embourgeoisement OR Déplac* OR Verdräng*)

Search date, number of results: June 11, 2025; **495**

## OATD

(agric* OR farm* OR garden* OR "food forest" OR agroforest* OR livestock OR husbandry OR beekeep* OR horticultur* OR greenhous* OR aquaponi* OR hydroponi* OR aeroponi* OR ferme* OR jardin* OR potager OR verger* OR elevage OR apicult* OR apicole* OR hortic* OR serre OR Aquacult* OR Landwirt* OR Anbau* OR Bauernh* OR Garten* OR Lebensmittelwald OR Agroforst* OR Vieh* OR Imker* OR Biene* OR Gärtner* OR Gewächsh* OR Treibh* OR Hydrokultur) AND (gentrif* OR displac* OR Embourgeoisement OR Deplac* OR Verdräng*)

Parameters:

- Simple search, reduced for platform functionality (removal of urban search string)
- Languages filtered individually in results: English, French, German

Search date, number of results: June 11, 2025; **1,338**

Notes: This database to be searched after all others to ease duplication checking, sorting by Author

## DissOnline (Deutsche National Bibliothek)

Parameters:

- Portal.dnb.de
- Expert search
- Material sort: Online Resources

((urban*) OR (city) OR (cities) OR (metro*) OR (commun*) OR (allotment*) OR (guerrilla) OR (vertical*) OR (indoor) OR (roof*) OR (zero acreage) OR (urbain*) OR (ville*) OR (collecti*) OR (familia*) OR (insertion*) OR (ouvrier*) OR (partagé*) OR (toit*) OR (hors-sol) OR (Stadt*) OR (Kommun*) OR (Kleingarten) OR (Schrebergarten) OR (Guerilla*) OR (Vertikale*) OR (Dach*)) AND ((agric*) OR (farm*) OR (garden*) OR (food forest) OR (agroforest*) OR (livestock) OR (husbandry) OR (beekeep*) OR (horticultur*) OR (greenhous*) OR (aquaponi*) OR (hydroponi*) OR (aeroponi*) OR (ferme*) OR (jardin*) OR (potager) OR (verger*) OR (Élevage) OR (apicult*) OR (apicole*) OR (hortic*) OR (serre) OR (serres) OR (Aquacult*) OR (Landwirt*) OR (Anbau*) OR (Bauernh*) OR (Garten*) OR (Lebensmittelwald) OR (Agroforst*) OR (Vieh*) OR (Imker*) OR (Biene*) OR (Gärtner*) OR (Gewächsh*) OR (Treibh*) OR (Hydrokultur)) AND ((gentrif*) OR (displac*) OR (Embourgeoisement) OR (Déplac*) OR (Verdräng*))

Search date, number of results: June 11, 2025; **35**

Notes: This database to be searched after all others to ease duplication checking
